# General classification of rhinopaties: the need for standardization according to etiology and nasal cytology

**DOI:** 10.1007/s00405-023-08117-3

**Published:** 2023-07-18

**Authors:** M. Gelardi, V. Fiore, R. Giancaspro, F. M. Di Canio, C. Fiorentino, S. Patruno, A. Ruzza, M. Cassano

**Affiliations:** Unit of Otolaryngology, Department of Clinical and Experimental Medicine, University of Foggia, Via Luigi Pinto 1, 71122 Foggia, Italy

**Keywords:** Rhinitis, Rhinopathies, Nasal cytology, Vasomotor rhinitis, Allergic rhinitis, Classification of rhinopathies

## Abstract

**Background:**

Rhinitis is as an inflammation of the nasal mucosa, characterized by high prevalence, widespread morbidity, and a significant financial burden on health care systems. Nevertheless, it is often considered as no more than a mere annoyance. This point of view has progressively led to underestimate and trivialize the disease. Therefore, there are numerous, mostly overlapping classifications of rhinopaties, but clear and standardized guidelines for diagnosis and treatment are still lacking. In the context of Precision Medicine, the development of a classification system focused on the endotypes of rhinitis to be widely adopted appears of utmost importance, also by virtue of study of the nasal immunophlogosis that, thanks to nasal cytology (NC), has recently allowed to better define the different forms of rhinitis, giving a new nosological dignity to several rhinopaties.

**Aim:**

We aimed to summarize the current knowledge regarding rhinitis and to propose a systematic classification of rhinitis, based on both etiology and cytological findings

## Introduction

Rhinitis is defined as an inflammation of the nasal mucosa, typified by the presence of one or more of the following: nasal congestion, pruritus, sneezing, rhinorrhea, and posterior nasal drainage. Based on etiology, rhinitis has been mainly characterized as infectious, allergic and, non-allergic [1]. Despite the high prevalence and widespread morbidity associated with a significant financial burden on health care systems, rhinitis is often considered as no more than a mere annoyance [2]. This point of view has lead over time to underestimate and trivialize the disease, hindering the development of clear guidelines for its diagnosis and treatment. As a matter of fact, there are numerous, mostly overlapping classification systems based on independent criteria, including age of onset, disease severity, symptoms, symptom pattern/frequency, causative agents, and underlying pathophysiology [3]. Thus, the development of a classification system focused on the endotypes of rhinitis to be widely adopted appears of utmost importance to standardize and correctly guide the diagnostic–therapeutic path of rhinitis. In this context, the study of the nasal immunophlogosis, thanks to the advent of nasal cytology (NC), has recently allowed to better define the different forms of rhinitis, giving a new nosological dignity to several rhinopaties, such as non-allergic rhinitis (NAR), which has been too long trivially considered a “non-specific” disease, given the unknown etiology [4]. This new cytological point of view has allowed to classify NAR, according to the predominant inflammatory cells infiltrating the nasal mucosa, into NARES (NAR with eosinophils), NARMA (NAR with mast cells), NARNE (NAR with neutrophils) and NARESMA (NAR with eosinophils and mast cells) [5]. Moreover, NC has allowed to define Overlapping rhinitis (ORs), in which allergic rhinitis (AR) and NAR coexist [6].

The aim of this review is to summarize the current knowledge regarding rhinitis and to propose a systematic classification of rhinitis, based on both etiology and cytological findings, with a view to standardizing the diagnostic–therapeutic path and guaranteeing patients effective and tailored treatments.

## General classification of rhinitis


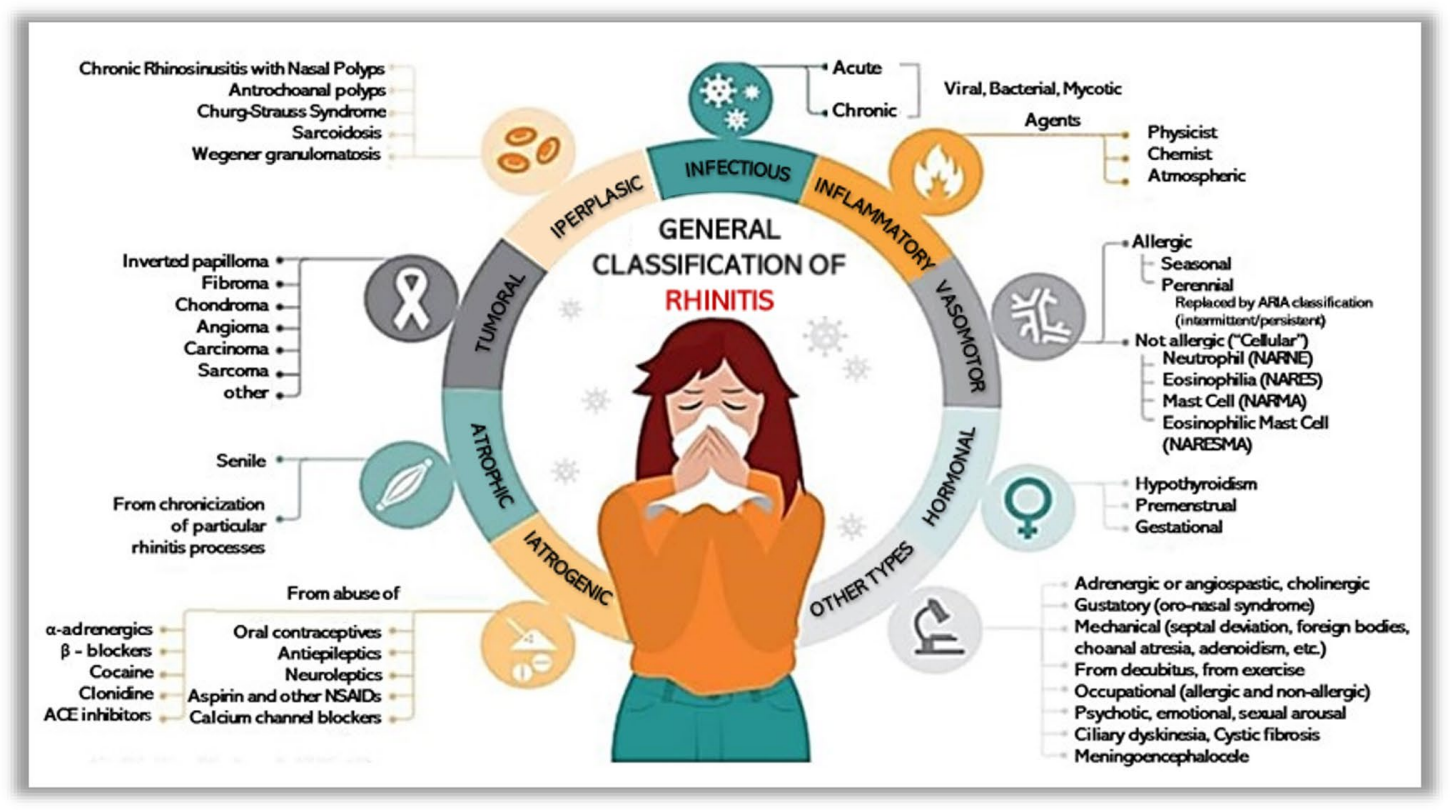


According to the etiology and the underling pathophysiologic mechanisms, rhinitis can be mainly classified as infectious, inflammatory, vasomotor, hormonal, iatrogenic, atrophic, tumoral, and hyperplastic. Moreover, other rarer forms of rhinitis can be associated with occupational factors, mechanical causes, ciliary dyskinesia, psychotic pathologies, and other less common causes. In turn, all these types of rhinitis can be further subclassified according to their specific characteristics.

In this context, NC allows to evaluate the well-being of the ciliated cells, the relationship between ciliated cells and goblet cells as well as the presence of immunophlogosis cells infiltrating the mucosa, to perform a precise diagnosis.

## Infectious rhinitis

Infectious rhinitis can be classified according to the pathogen as bacterial, viral, and fungal or according to the duration as acute and chronic. NC allows to visualize bacteria and fungi in NC samples, as well as the alterations induced by viral infections, leading to a diagnosis of infectious rhinitis, even without performing a culture test by nasal swab.

**Fig. 1:**
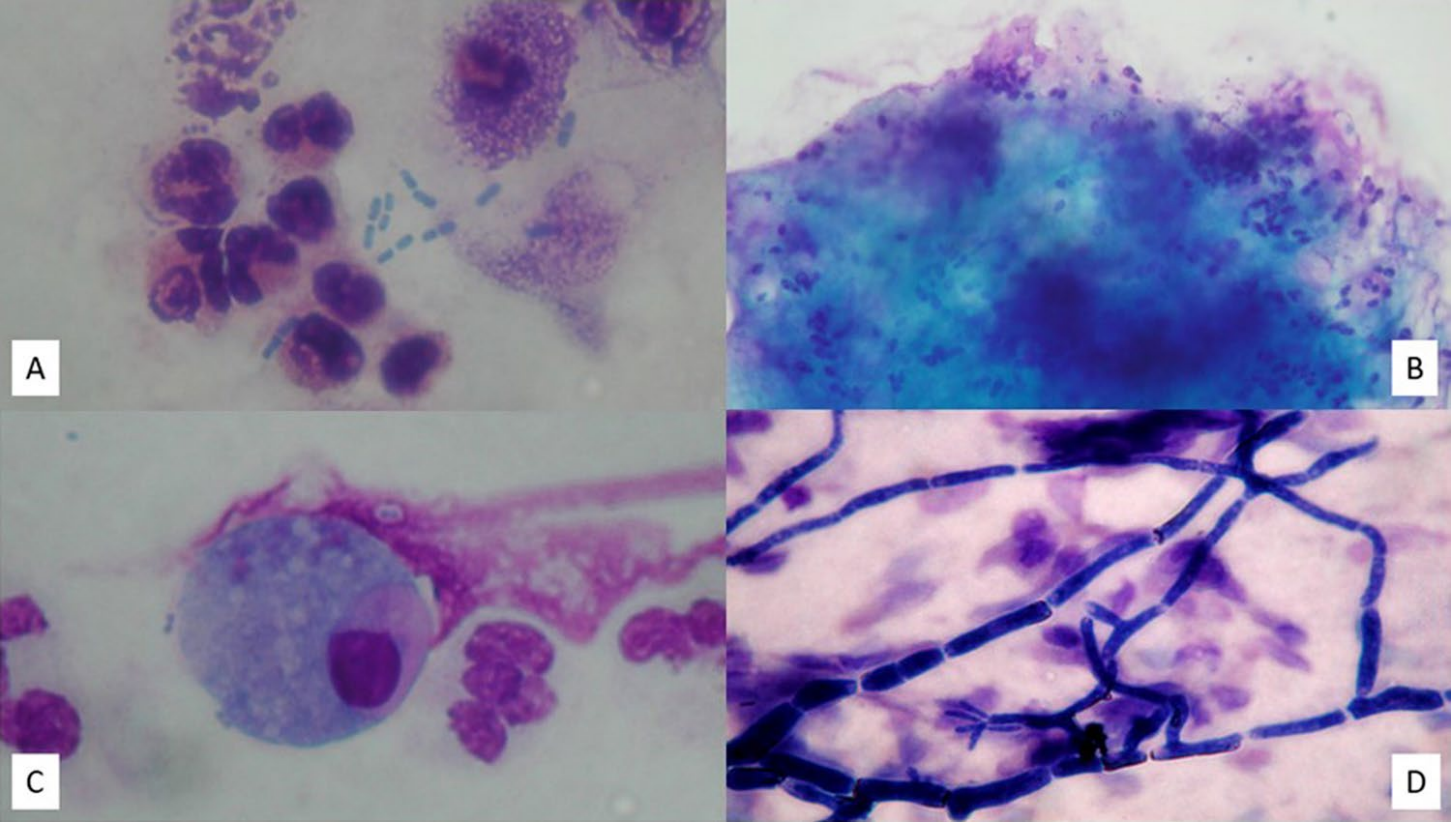
Nasal cytology of different forms of infectious rhinitis. MGG staining. Magnification 1000×. **A** Bacterial rhinitis characterized by numerous neutrophils and bacteria in planktonic form. **B** Bacterial biofilm. **C** Viral rhinitis, characterized by Ciliocytophthoria. **D** Fungal rhinitis, showing fungal hyphae

In the case of bacterial rhinitis, numerous resident or circulating cell populations appear on the surface of the respiratory mucosa, such as neutrophils, lymphocytes, and macrophages, and flaking epithelial cells increase. From a microscopic point of view, the nasal cytological samples of patients suffering from bacterial rhinitis show numerous neutrophils and various microorganisms, including bacteria adherent to the cell surface or inside the phagocytes (Fig. 1a). Furthermore, typically, the hair cells decrease in number in favor of goblet and metaplastic cells, and the infectious stain, which is morphological–chromatic expression of the biofilm, appears (Fig. 1b) [7].

Often, bacterial rhinitis follows viral rhinitis, by virtue of the cytopathic effect of viruses on the mucous epithelium. The latter type of rhinitis is characterized by edema and congestion of the nasal mucosa, which is covered with a seromucous exudate. From a microscopic point of view, surface epithelial cells undergo a series of morphological modifications, known as Ciliocytophthoria (Fig. 1c). It is worth mentioning that in viral rhinitis the regeneration processes of the epithelium lead to *restitutio ad integrum*, at the end of the infection, unlike bacterial inflammations, which frequently cause scarring [8]. Interestingly, by virtue of the recent pandemic from COVID-19, the attention of the research has focused on the identification of the cytopathic effect caused by the Severe Acute Respiratory Syndrome Coronavirus 2 (SARS-CoV-2), which seems to have a particular tropism right on the ciliated cells of the nasal mucosa [9, 10].

Fungal rhinitis, on the other hand, is a rarer infectious rhinitis, which mainly affects immunocompromised subjects. The cytopathic effects of the fungi mainly occur in the nuclei, which appear rarefied. In addition, spores or fungal infections can invade the cytoplasm and nucleus of cells (Fig. 1d).

## Inflammatory rhinitis

**Fig. 2:**
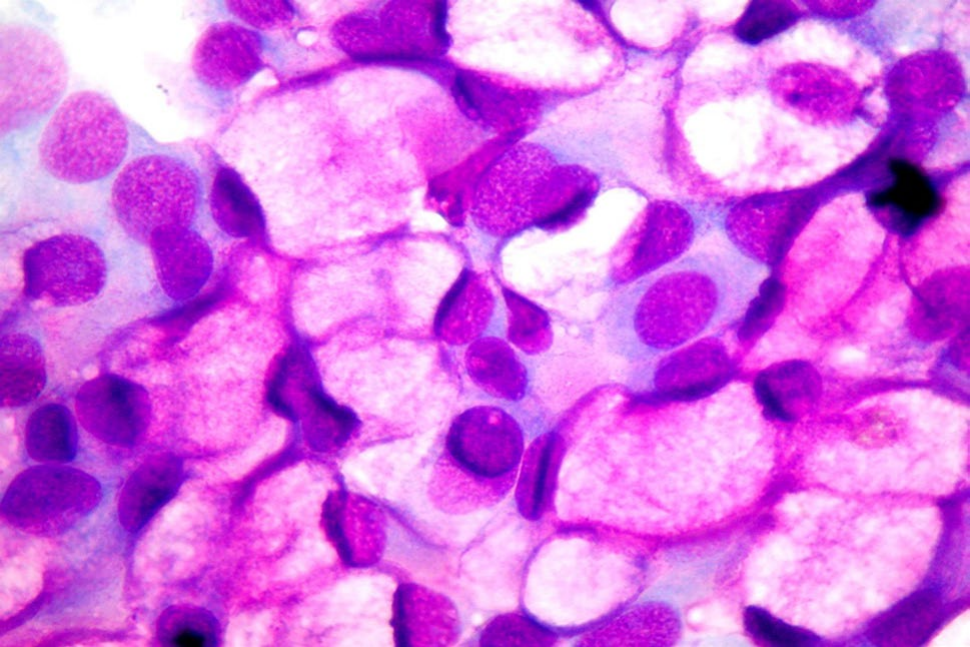
Nasal cytology of inflammatory rhinitis, characterized by muciparous metaplasia. MGG staining. Magnification ×1000

Inflammatory rhinitis is associated with continuous exposure to physical, chemical, or atmospheric irritants, which progressively compromise the integrity of the mucosa lining the airways and generate a vicious circle responsible for the histolesive process. Both the ciliodyskinetic action of irritants and the chronic inflammation itself compromise mucociliary clearance, favoring bacterial colonization and the onset of infections. In particular, in heavy smokers the cells of the respiratory epithelium undergo intense flaking and morphological modifications. In addition, in subjects exposed to unfavorable environmental conditions, such as excessive temperature excursions, fumes or dust, the number of goblet cells increases [11]. NC highlights mucosal alterations caused by chronic inflammation, represented by epithelial metaplasia, which can be proliferating, with an increase in muciparous cells (muciparous metaplasia) (Fig. 2) or atrophic, with an increase in squamous cells (platicellular metaplasia) [12].

## Vasomotor rhinitis

Allergic rhinitis (AR) and non-allergic rhinitis (NAR) belong to the large category of vasomotor rhinitis. In particular, the term vasomotor refers to nasal hyper-reactivity, that is the symptomatic expression of the capacity of nasal mucosa to respond to allergenic or nonspecific stimuli, such as temperature, humidity, and odors.

Before the introduction of NC among rhinological investigation, non-allergic forms were defined as "non-specific", since an IgE-specific sensitization was excluded. Nevertheless, thanks to NC, these forms have gained a nosological dignity and are now classified according to the predominant cell types into NARES (NAR with eosinophils), NARMA (NAR with mast cells), NARNE (NAR with neutrophils) and NARESMA (NAR with eosinophils and mast cells) [13]. Therefore, nowadays, it is correct to define this type of rhinitis as vasomotor, specifying whether allergic or non-allergic and, in the latter case, specifying the subtype according to the predominant inflammatory cells.

### Allergic rhinitis

AR is an inflammatory disorder of the nasal mucosa induced by allergen exposure triggering IgE-mediated inflammation [14]. Traditionally, AR has been classified based on the time of allergen exposure into seasonal, when occurring during a specific season, or perennial, when occurring throughout the year. While useful, this classification posed several problems, since it is difficult to differentiate between seasonal and perennial symptoms, the exposure to some pollen allergens is long standing and not similar over the year, the nasal inflammation is prolonged for weeks after pollen exposure in patients with seasonal rhinitis and the majority of patients are polysensitized to pollen and perennial allergens [15]. Therefore, Allergic Rhinitis and its Impact on Asthma (ARIA) Guidelines have introduced a new classification and AR is nowadays classified into “intermittent”, when symptoms present less than 4 days per week or for less than 4 consecutive weeks, or “persistent”, when symptoms are present more than 4 days/week and for more than 4 consecutive weeks [15]. Moreover, symptoms are classified as mild when patients have no impairment in sleep and are able to perform normal activities or moderate/severe if they significantly affect sleep or activities of daily living, and/or if they are considered bothersome [16].

**Fig. 3:**
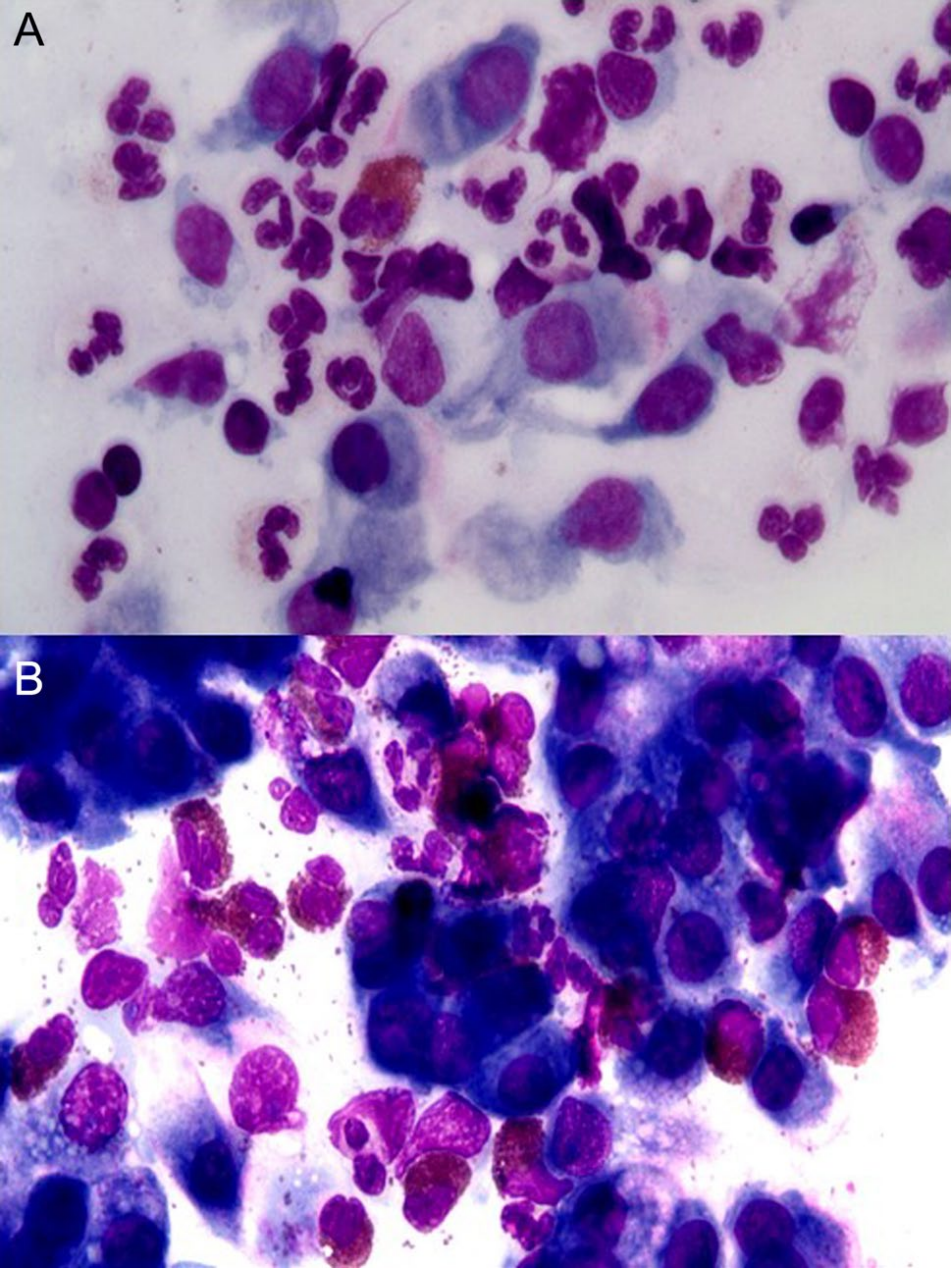
Nasal cytology of AR. MGG staining. Magnification 1000×. **A** Rhinocytogram of a patient suffering from perennial AR, characterized by minimal persistent inflammation. **B** Rhinocytogram of patient suffering from intermittent AR, during the pollen period, characterized by massive eosinophilic–mast cell degranulation

Patients suffering from AR, when stimulated naturally or by specific nasal provocation tests, develop an allergic reaction which consists of a so-called early phase, mainly mediated by histamine, and a late phase, caused by inflammatory cells. From a cytological point of view, these responses are characterized by the presence of inflammatory cells infiltrating the nasal mucosa, including eosinophils, mast cells, neutrophils, and plasma cells, which release several chemical mediators, responsible for the main symptoms of AR (itching, nasal congestion, runny nose, sneezing, etc.) [17]. NC findings vary according to the type of allergens to which patients are sensitive. Indeed, rhinocytograms of patients suffering from perennial AR, characterized by an allergen exposure of low intensity but persistent in time, show “minimal persistent inflammation” that consist in a persistent infiltration of neutrophils and only minimally by eosinophils (Fig. 3a). On the contrary, rhinocytograms of patients with seasonal AR change depending on whether patients are examined during the exposure of allergens or not. During the pollen season, patients present all the clinical signs and symptoms of AR and NC show neutrophils, lymphocytes, eosinophils and MCs, largely degranulated (Fig. 3b), while out of season of exposure, they present a clinical and cytological “silence” [18].

### Non-allergic rhinitis

**Fig. 4:**
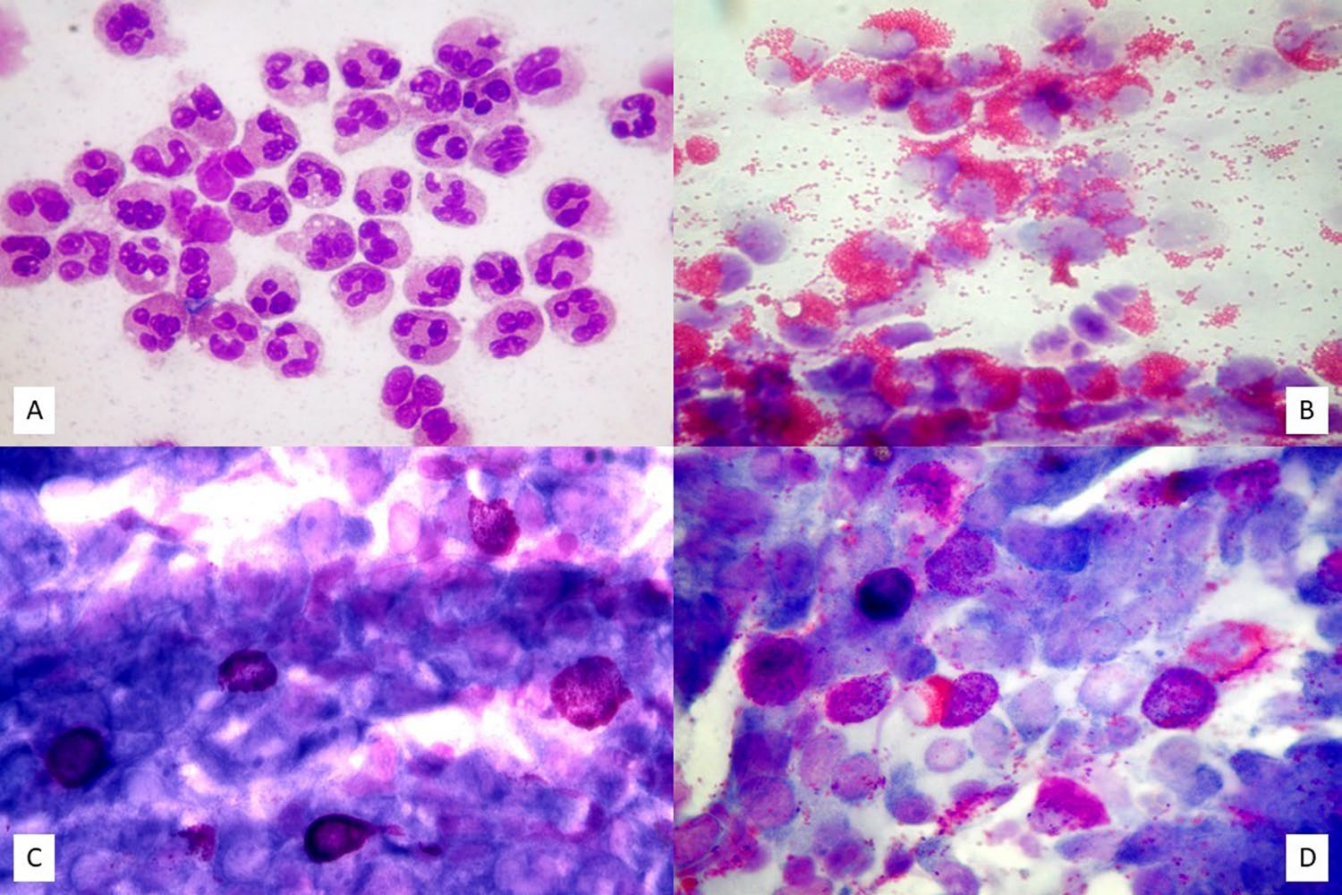
Nasal cytology of NAR. MGG staining. Magnification ×1000. **A** NARNE. **B** NARES. **C** NARMA. **D** NARESMA

NARs, also defined as “cellular rhinitis”, have a non-univocal clinical–diagnostic–therapeutic approach and, for this reason, have often been misdiagnosed before the advent of NC.. As a matter of fact, these rhinitis represent approximately 15% of all rhinopaties and are usually characterized by intense pseudo-allergic symptoms, which is why they are often confused as IgE-mediated rhinitis and generally defined as non-specific. The latter definition must be abolished, because these rhinopaties have to be classified, according to the cytotype most represented in NC, as NARNE, NARES, NARMA, NARESMA (Fig. 4).

### NARNE

NARNE is characterized microscopically by an infiltration of neutrophils (> 30%), in the absence of bacteria, and fungal spores or hyphae. The incidence of this rhinopathy has increased in recent years, probably due to industrialization and increased smoking habits, since the majority of patients suffering from this form of rhinitis are industrial workers, smokers or people living in industrialized areas [19]. Gastroesophageal reflux is one of the causes of NARNE, as exhaling hydrochloric acid and its contact with the nasal mucosa attracts neutrophils, first response inflammatory cells, to the mucosal level [20]. The permanence of these cells and the release of chemical mediators, including neutrophilic elastase, leads to the formation of free radicals and to mucosal damage, which result in "vasomotor" symptoms, such as sneezing, nasal congestion and seromucous rhinorrhea [21]. Among NAR, NARNE it is not a congenital pathology, it can regress once the pathogenic cause has been removed and it is characterized by symptoms.

### NARES

NARES is a vasomotor non IgE-mediated rhinitis, characterized by an abundant eosinophilic infiltration of the nasal mucosa, generally up to 50–70% [13]. Sometimes, the abundant local eosinophilic inflammation can be associated with blood hypereosinophilia.

As for NARMA and NARESMA, NARES is typically associated with Chronic Rhinosinusitis with nasal polyps (CRSwNP), ASA sensitivity and asthma. Furthermore, the eosinophils infiltrating the nasal mucosa can in turn recruit mast cells, through the production of numerous cytokines and chemokines, effectively evolving into eosinophilic–mast cell forms of rhinitis (NARESMA), characterized by more intense and continuous symptoms.

### NARMA

NARMA is characterized by the presence of mast cells, partially degranulated, in NC samples and can be associated with systemic mastocytosis [22]. Symptoms associated with this type of rhinitis, such as nasal obstruction, rhinorrhea, and sneezing, are typically very intense. Moreover, NARMA can evolve into NARESMA and can be associated with asthma and CRSwNP.

### NARESMA

NARESMA is a recently described type of cellular rhinitis, characterized by abundant eosinophils and mast cells, partially degranulated. NARESMA, more than all other forms of NAR, it tends to be associated with CRSwNP and asthma as well as with a worsening of patients' Quality of Life (QoL). Furthermore, if associated with CRSwNP, NARESMA constitutes an unfavorable prognostic index of recurrence [5].

## Overlapping rhinitis

NC has recently brought to light an important problem: since AR and NAR are two separate entities, they can coexist in the same patient in up to 15–20% of cases. Overlapping rhinitis (ORs) are considered traps for allergists and otolaryngologists, since they are often misdiagnosed, resulting in the failure of medical and surgical strategies. Indeed, from a clinic point of view, patients with OR are often treated as pure allergic, even if they present an intense symptomatology outside the pollen period. NC findings of these patients are characterized by eosinophils and/or mast cells, even when not exposed to specific allergens. Since the therapeutic strategies target specifically allergic processes, patients with OR experience only a partial benefit, despite expectations. This emphasizes the importance of identifying, with the aid of the NC, the specific pathology of which patients are affected, since only a correct diagnosis can guarantee an effective therapy [6].

## Hormonal rhinitis

Gestational rhinitis belongs to the group of hormonal rhinitis and has a prevalence between 9% and 22%. This rhinitis typically appears in the second and third trimester of pregnancy and undergoes complete resolution of symptoms within 2 weeks of delivery. From the pathophysiological point of view, the pregnancy hormonal corollary seems to be involved, since estrogen and progesterone are associated with rhinorrhea and mucosal hypertrophy [23]. Even the hormones of the menstrual cycle can affect the nasal mucosa, causing vasodilatation, edema, and increased secretion by increasing the expression of histamine H1 receptors and altering the concentration of neurotransmitters, such as substance P. Since the obstructive symptoms are directly associated with vasodilatation, no important changes in the nasal mucosa are observed on nasal cytology, except for a muciparous metaplasia, which is due to an increase in mucus secretion induced by the alteration of neurotransmitter concentrations. The same cytological alteration can be find in another type of rhinitis that related to hormonal imbalances, such as that associated with hypothyroidism. This rhinopathy is directly related to the increase in the Thyroid-stimulating hormone (TSH), which can cause the hypertrophy of the mucous glands, the increase in the submucosal connective tissue due to the deposition of acid mucopolysaccharides and the destruction of the mucous ciliary apparatus [24].

## Iatrogenic rhinitis

Iatrogenic rhinitis can be related both to the chronic use of nasal vasoconstrictors and to the abuse of narcotic substances, such as cocaine, as well as to systemic drugs that also exert their action on the nasal mucosa, such as β-adrenergics, α-blockers and oral contraceptives. This type of rhinitis is characterized by no variations in the cellular component at NC, unless concomitant allergic or infectious pathologies are associated [25].

## Atrophic rhinitis

Atrophic rhinitis is a chronic rhinitis characterized by atrophy of the nasal mucosa and submucosa. The improvement of living and health conditions has reduced the incidence of this form of rhinitis over the years. Since atrophic rhinitis mostly affects young women, the causal agent is hypothesized to be hormonal in nature, although viruses and immune deficiencies can also trigger the pathology. Clinically, patients present with atrophic turbinates and abnormally large nasal cavities filled with crusted exudate [26]. From the cytological point of view, the mucosa undergoes squamous metaplasia of various degrees with chronic inflammatory infiltrate.

Atrophic rhinitis can also be found in elderly subjects, in whom the mucous epithelium undergoes progressive atrophy, with reduction of goblet cells and increase of squamous cells.

## Hyperplastic/granulomatous rhinitis

Chronic rhinosinusitis with nasal polyps (CRSwNP), affecting approximately 4% of the general population, is a chronic inflammatory disease of the nasal mucosa, characterized by the presence of cardinal symptoms, such as nasal obstruction, rhinorrhea, facial pain and hyposmia or anosmia lasting longer than 12 weeks. CRSwNP is characterized by the presence of nasal polyps, which are translucent, pale, gelatinous protrusions of the nasal mucosa that in most cases originate in correspondence with the middle meatus and anterior ethmoid region [27]. From a histological point of view, they are characterized by an edematous support structure covered by ciliated pseudostratified epithelium, which is infiltrated by numerous inflammatory cells, including eosinophils, mast cells and neutrophils. In fact, CRSwNP is believed to be a hyperplastic-degenerative expression of the nasal mucosa, secondary to a chronic "cellular" rhinopathy, such as NARES, NARMA and NARESMA. However, due to limitations in magnification and staining, histological examination does not allow visualization of all cytotypes infiltrating the nasal mucosa. In this context, NC not only allows to highlight all cytotypes of nasal immunophlogosis but also to establish the severity of CRSwNP, according to Clinical–Cytological Grading (CCG). In particular CCG assesses the severity of the disease as a function of NC findings and comorbidities, each of which corresponds to a specific score. The final CCG score corresponds to the sum of the scores attributed to the endotype (neutrophilic infiltrate corresponds to 1, mast cell to 2, eosinophilic to 3, mixed eosinophilic–mast cell to 4), represented by the predominant cellular infiltrate, and to the phenotype, represented by the various comorbidities (ASA sensitivity corresponds to 1, asthma to 2, allergy to 3, ASA sensitivity combined to asthma to 3). Since the severity of the disease is strictly correlated with the refractoriness of the disease, the CCG corresponds to the Prognostic Index of Relapse (PIR), which is properly the prognostic expression of CCG [28].

Granulomatous/hyperplastic forms include antrochoanal polyps (ACP), which are benign polypoid lesions originating from the inner wall of the maxillary sinus and extending into the choana. The inflammatory pattern of the ACP is predominantly Type 1 neutrophilic. In addition to the inflammatory component, from a microscopic point of view, APC are characterized by the presence of tissue remodeling, mainly fibrin deposition and edema, and cysts in the epithelium and lamina propria [29].

Furthermore, Sarcoidosis and systemic vasculitis such as Wegener granulomatosis and Churg–Strauss syndrome can be associated with hyperplastic rhinitis.

## Tumoral rhinitis

**Fig. 5:**
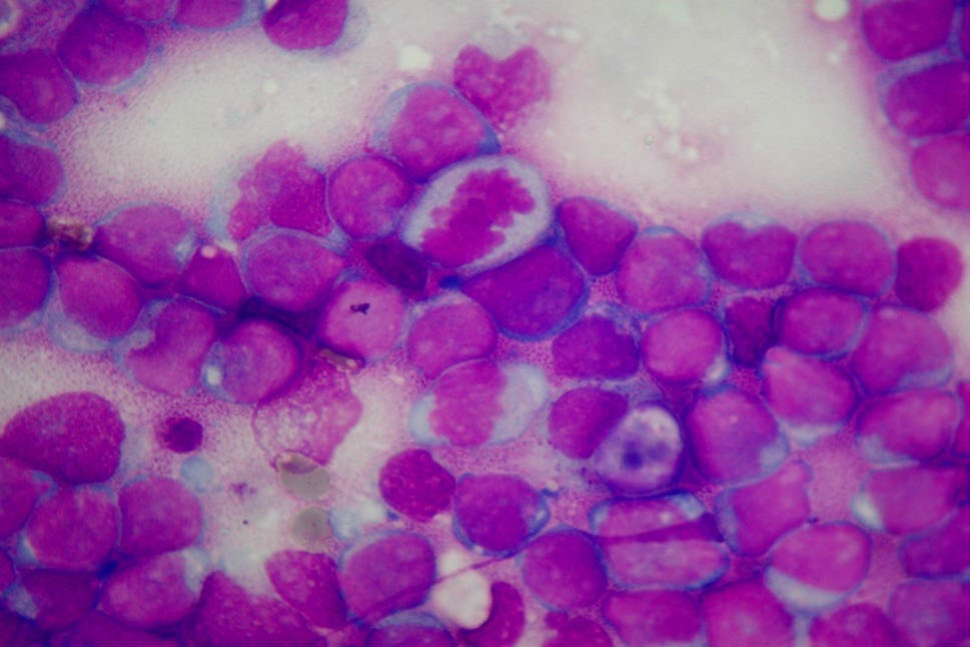
Nasal Cytology. MCC staining. Magnification ×1000. Massive lymphocytic infiltrate, characterized by abnormal cytopathological features, including irregular nuclei, numerous mitotic figures, dyschromic chromatin and compacted chromatin at the periphery of the nuclei

Rhinitis can be related to different types of tumors, including carcinomas, sarcomas. Recently, an association between rhinitis and leukemia has been demonstrated. Indeed, since the cytological characteristics of the infiltrating inflammatory cells reflect any cytopathological alterations of the blood circulating cells, findings of patients affected by oncohematological diseases could also be found in nasal inflammatory infiltrate (Fig. 5). This conspicuous lymphocytic infiltrate in turn can caused vasomotor rhinitis [30]. On the contrary, no alteration can be found in the NC of patients affected by tumors of the nose or paranasal sinuses, since the cytological sampling is performed on the inferior turbinate and not on the neoplastic tissue level. Therefore, such patients may present with obstructive symptoms, possibly associated with rhinorrhea, with normal NC.

## Other rhinitis

Other rhinitis include angiospastic rhinitis, which is associated with sympathetic–tonic syndromes, such as acrocyanosis and Raynaud's syndrome, and cholinergic vasomotor rhinitis, associated with acetylcholine release due to vagal arrest. Moreover, among other causes of rhinitis, the is the abnormal gustatory reflex associated with a hyperactive, nonadrenergic, noncholinergic, or peptidergic neural system, causing a gustatory rhinitis, a conspicuous type of food-associated rhinorrhea, which can occasionally be associated with significant quality-of-life [31]. Even mechanical causes, such as nasal septal deviation, choanal atresia, the presence of foreign bodies, as well as decubitus, physical exercise, occupational and psychological factors, pathologies such as ciliary dyskinesia, cystic fibrosis and myelomeningocele can be involved in the pathogenesis of different rhinitis. In these types of rhinitis, specific NC alterations are not described. Therefore, for a correct diagnosis, it is essential to evaluate not only the NC, but also the pathological history, the work history and any risk factors.

## Conclusions

Despite the high prevalence and widespread morbidity of rhinopaties, a general and uniform classification of rhinitis is still lacking. In this context, the advent of NC has recently made it possible to clarify the general aspects of the different rhinitis, allowing the creation of a classification, based on both etiology and cytological findings. In fact, standardizing the diagnostic–therapeutic pathway appears to be of fundamental importance, above all by virtue of the evolution of specific rhinitis, such as NARES and NARESMA, towards CRSwNP. In this perspective, NC and an accurate anamnestic collection, aimed at evaluating each comorbidity and risk factor, are complementary and both indispensable steps for making an accurate rhinological diagnosis.

We hope that this review will allow a unified diagnostic path to rhinitis, to allow the homogeneity of the clinical approaches and the realization studies aimed at standardizing the therapeutic strategies as well, thus reducing the economic and social burden not only of rhinitis but also of as CRSwNP.
